# Neonicotinoid Insecticide Imidacloprid Causes Outbreaks of Spider Mites on Elm Trees in Urban Landscapes

**DOI:** 10.1371/journal.pone.0020018

**Published:** 2011-05-31

**Authors:** Adrianna Szczepaniec, Scott F. Creary, Kate L. Laskowski, Jan P. Nyrop, Michael J. Raupp

**Affiliations:** 1 Department of Entomology, University of Maryland, College Park, Maryland, United States of America; 2 Department of Entomology, Cornell University, Ithaca, New York, United States of America; AgroParisTech, France

## Abstract

**Background:**

Attempts to eradicate alien arthropods often require pesticide applications. An effort to remove an alien beetle from Central Park in New York City, USA, resulted in widespread treatments of trees with the neonicotinoid insecticide imidacloprid. Imidacloprid's systemic activity and mode of entry via roots or trunk injections reduce risk of environmental contamination and limit exposure of non-target organisms to pesticide residues. However, unexpected outbreaks of a formerly innocuous herbivore, *Tetranychus schoenei* (Acari: Tetranychidae), followed imidacloprid applications to elms in Central Park. This undesirable outcome necessitated an assessment of imidacloprid's impact on communities of arthropods, its effects on predators, and enhancement of the performance of *T. schoenei*.

**Methodology/Principal Findings:**

By sampling arthropods in elm canopies over three years in two locations, we document changes in the structure of communities following applications of imidacloprid. Differences in community structure were mostly attributable to increases in the abundance of *T. schoenei* on elms treated with imidacloprid. In laboratory experiments, predators of *T. schoenei* were poisoned through ingestion of prey exposed to imidacloprid. Imidacloprid's proclivity to elevate fecundity of *T. schoenei* also contributed to their elevated densities on treated elms.

**Conclusions/Significance:**

This is the first study to report the effects of pesticide applications on the arthropod communities in urban landscapes and demonstrate that imidacloprid increases spider mite fecundity through a plant-mediated mechanism. Laboratory experiments provide evidence that imidacloprid debilitates insect predators of spider mites suggesting that relaxation of top-down regulation combined with enhanced reproduction promoted a non-target herbivore to pest status. With global commerce accelerating the incidence of arthropod invasions, prophylactic applications of pesticides play a major role in eradication attempts. Widespread use of neonicotinoid insecticides, however, can disrupt ecosystems tipping the ecological balance in favor of herbivores and creating pest outbreaks.

## Introduction

One of the most ecologically significant outcomes of global change is the rapid redistribution of biota [1]. The number of alien insect species introduced to the United States has increased dramatically during the last two centuries [2] and alien species account for more than $120 billion in damage annually in the United States [3]. Recently, two alien wood-boring beetles, the Asian longhorned beetle, *Anoplophora glabripennis*, and the emerald ash borer, *Agrillus planipennis* have killed tens of millions of trees and dramatically altered the composition of forests in cities and natural lands in North America [4], [5].

To halt these rapidly spreading beetles in the United States, federal quarantine and eradication programs destroyed thousands of infested trees and applied insecticides to protect tens of thousands of others [4], [6], [7]. One insecticide widely used in eradication efforts is imidacloprid. Imidacloprid belongs to a relatively new class of insecticides, the neonicotinoids. These nitroguanidine compounds have impressive toxicity against a wide range of economically important pests [8] and long residual activity [9]–[12]. Unlike nicotine, neonicotinoids exhibit a selective affinity for nerve cell receptors of insects [13]. Their broad spectrum of activity kills many insect pests, but one economically important family of herbivorous arachnids, spider mites (Tetranychidae), are insensitive to neonicotinoids [8] and their abundance may increase following imidacloprid applications [9], [14], [15].

Following the discovery of *A. glabripennis* in Central Park, New York, in 1996, an effort to eradicate the pest and save historically important trees including a stand of American elms, *Ulmus americana*, resulted in more than 14,000 applications of imidacloprid between 2005 and 2007 [16]. This intensively managed ecosystem provided a unique opportunity to investigate the effects of imidacloprid on community structure of arboreal arthropods, in particular on population dynamics of spider mites on elm trees and physiological responses of non-target arthropods to imidacloprid exposure. Changes in the structure and function of arthropod communities following pesticides applications have been examined in several agricultural [17]-[20] and aquatic [21]–[24] systems, but rarely in urban ecosystems [25], [26]. Results of terrestrial studies in agriculture revealed significant shifts in arthropod communities characterized by reductions in richness, diversity, density, and biomass of many arthropod species following pesticide applications [17]–[20]. Similar changes have been documented in aquatic communities following exposure to insecticides [21]–[23]. Moreover, pesticides restructure heterospecific interactions including competition and predation in invertebrate communities [24].

While pesticides reduce abundance and mitigate the impact of invasive pests, they also disrupt ecological processes resulting in outbreaks of pests and reductions in yields or quality of crops [27]. Early mechanistic explanations for resurgences of primary pests (targets of pesticide applications) and outbreaks of secondary pest (pests not targeted by applications) focused on pesticide-driven elimination of predatory arthropods, which play a major top-down role in suppressing herbivores in managed ecosystems [25]–[28]. Recently, complementary mechanisms have been used to explain increases in pest populations following pesticide applications [27], [29]. These include hormoligosis or hormesis [27], [29]–[31], defined as elevated fecundity of herbivores following sub-lethal exposure to pesticides. Additionally, some insecticide classes such as neonicotinoids may alter physiological pathways within plants [14], [27], [29], [32], [33] and improve their nutritional value for herbivores [14], [29], [32].

Here we report changes in the structure of arboreal arthropod communities and in the populations of arthropods following applications of imidacloprid in Central Park, New York and College Park, Maryland. The study site in New York received imidacloprid applications as part of a federally mandated quarantine and eradication effort and randomization of treatments was restricted by law. A common garden study site was established in Maryland to permit randomization of treatments and examine more thoroughly changes in the arthropod community and populations of arthropods in response to applications of imidacloprid. Moreover, to elucidate mechanisms underlying changes in spider mite abundance documented by earlier published reports [9], [14], [15], in laboratory bioassays, we examined direct and indirect effects of imidacloprid on *Tetranychus schoenei* (Acari: Tetranychidae), the most abundant spider mite on elm trees in New York and Maryland, and two model insect predators, *Stethorus punctillum* and *Chrysoperla rufilabris*. Other studies examined independently effects of imidacloprid on populations of other species within Tetranychidae [9], [14], [15], plant quality [14], [32], [33], and natural enemies [15], [34]–[42]. Our research, however, provides the first report of imidacloprid's impact on arthropod communities in urban landscapes and explores multiple mechanisms underlying changes in structure of arthropod fauna and outbreaks of spider mites. This is also the first study to separate the direct impact of imidacloprid on spider mite fecundity from the plant-mediated effect on spider mite reproduction. We show that imidacloprid enhances quality of plants for spider mites that lay more eggs when feeding on imidacloprid-treated plants. Furthermore, the importance of this research is underscored by the rate at which extensive trade exchange, increasing globalization, and climate change exacerbate pest problems [1], [2], [43] and the fact that insecticides like imidacloprid will be used to mitigate problems caused by alien pests [4]–[6].

## Results

### Preliminary surveys of arthropods in a quarantine zone in New York and effects of imidacloprid on arthropod communities in a common garden study in Maryland

**Figure 1:**
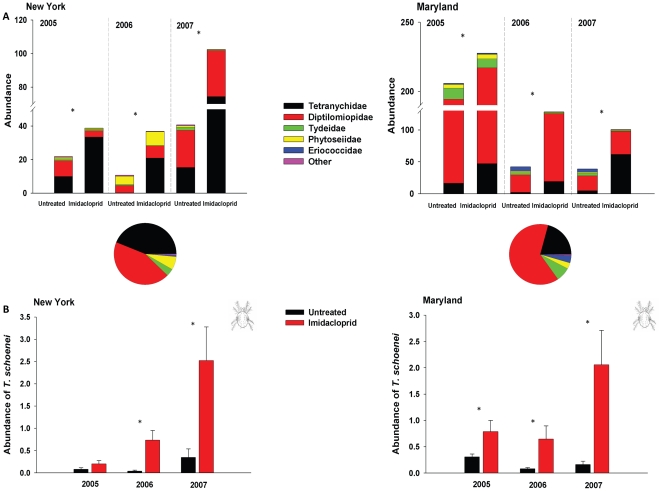
Effect of imidacloprid on arthropod communities and spider mite abundance in New York and Maryland. (A) Abundance of arthropods (per cm^2^ of leaf area) on imidacloprid-treated elms (*N* = 10) and on untreated trees (*N* = 10). Asterisks mark differences in overall abundance of arthropods that were significant within each year (*P*<0.05; Monte Carlo permutation test). At both locations, arthropod communities increased on elms that received imidacloprid. Abundance of spider mites, Tetranychidae, explained most of the variation due to imidacloprid treatments. Pie charts represent percent contribution of the most abundant taxa to the sampled arthropod community over three-year period at each location. (B) Abundance (√(number)/cm^2^) of the spider mite, *T. schoenei,* on elms treated with imidacloprid (*N* = 10) and on untreated trees (*N* = 10) in New York and Maryland. Asterisks mark means±s.e.m. that differed significantly (*P*<0.05). There was a significant interactive effect of treatment and time for both locations and in most years, and means were compared within each date (Table S1). Elevated densities of mites were found only on elms treated with imidacloprid. Rarely encountered taxa included arthropods in families Chrysopidae, Coccinellidae, Cecidomyiidae, Aphididae, Saproglyphidae and Thripidae. These arthropods were pooled and categorized as ‘Other’.

Owing to the fact that a federal agency mandated exactly which trees were treated with imidacloprid in Central Park, the random assignment of treatments was restricted. Therefore, inferences from preliminary surveys conducted in New York are interpreted conservatively and restricted only to trees in New York. Over the three years of this study, more than 254,990 arthropods were collected from the canopies of elms in New York and Maryland. Arachnids dominated communities of arboreal arthropods at both locations (Figure 1A). The less abundant taxa, grouped into ‘Other’ category, included Aphididae (aphids), Saproglyphidae (scavenger mites), Chrysopidae (green lacewings), Cecidomyiidae (predatory midges), Thripidae (thrips) and Coccinellidae (lady beetles in the genus *Stethorus*). Each taxon in this category was comprised of a small number of species, usually one or two, and did not differ significantly between trees treated with imidacloprid and untreated elms at either site. Eggs of Phytoseiidae and Chrysopidae were enumerated in addition to active stages of the predators, and eggs comprised 51% and 47% of all Phytoseiidae and Chrysopidae, respectively. We also captured other arthropods that did not account for more than 0.05% of all arthropods such as Anthocoridae, Araneae, Miridae, Reduvidae, and lepidopteran larvae among others.

The first ordination accounted for 10 to 20% of the observed variation in New York and 6 to 18% of the observed variation in Maryland over the course of the study. Imidacloprid significantly altered the structure of arthropod communities at both locations in each year of the study (New York: 2005, *F* = 11.27, *P*<0.001; 2006, *F* = 16.10, *P*<0.001; 2007, *F* = 18.89, *P*<0.001; Maryland: 2005, *F* = 11.95, *P*<0.001; 2006, *F* = 16.58, *P*<0.001; 2007, *F* = 16.50, *P*<0.001) (Figure 1A). With the exception of the first season in New York, *T. schoenei,* were far more abundant on imidacloprid-treated elms at both locations (New York: 2005, χ*^2^* = 0.13, df = 1, *P* = 0.722; 2006, χ*^2^* = 8.72, df = 1, *P* = 0.003; 2007, χ*^2^* = 3.61, df = 1, *P* = 0.051; Maryland: 2005, χ*^2^* = 5.09, df = 1, *P* = 0.024; 2006, χ*^2^* = 9.15, df = 1, *P* = 0.003; 2007, χ*^2^* = 10.86, df = 1, *P* = 0.001) (Figure 1B). Within most years, significant interactive effects of time and treatment on *T. schoenei* abundance were evident at both locations (New York: 2005, *F*_4,89_ = 7.27, *P*<0.001; 2006, *F*_2,54_ = 3.61, *P* = 0.011; 2007, *F*_3,72_ = 11.57, *P*<0.001; Maryland: 2005, *F*_4,85_ = 1.93, *P = *0.049; 2006, *F*_2,53_ = 0.5, *P = *0.737; 2007, *F*_2,54_ = 4.81, *P = *0.002). At the beginning of each growing season, *T. schoenei* were equally abundant on treated and untreated trees, but as the season progressed, *T. schoenei* became far more abundant on trees treated with imidacloprid (Figure S1, Table S1). In New York, differences in numbers of *T. schoenei* between treatments waned every fall (Figure S1). Other mites such as the omnivorous tydeids, *Homeopronematus anconai* and *Lorryia* sp., were more abundant on untreated trees in most years (Figure 1A, Table S2). *H. anconai* represented approximately 90% of the tydeid mites collected on all elm trees in New York and Maryland. Predatory phytoseiid mites (all in the genus *Galendromus*), were more abundant on untreated elms in 2007 in New York and in 2006 and 2007 in Maryland (Figure 1A, Table S2). Herbivorous mites in the family Diptilomiopidae, on the other hand, were more abundant on imidacloprid-treated elms in two out of three years in Maryland (Figure 1A, Table S2). Imidacloprid applications suppressed populations of the scale insect, *Gossyparia spuria* (Hemiptera: Eriococcidae) on elm trees in Maryland (Table S3). All other arthropods did not contribute significantly to differences in community structure on imidacloprid-treated and untreated elms (Table S4).

### Mechanisms underlying outbreaks of *T. schoenei*: Deleterious effects of imidacloprid on two model insect predators

**Figure 2:**
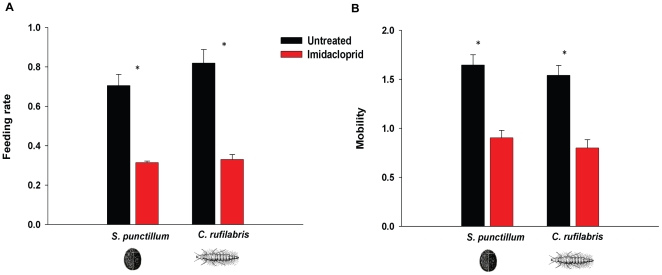
Feeding rate and mobility of *S. punctillum* and *C. rufilabris* exposed to imidacloprid through prey. A) Feeding rates (√(mites eaten)/h) of *S. punctillum* and *C. rufilabris* were reduced after 3.5 h when mites were reared on foliage from imidacloprid-treated (*N* = 7) compared to untreated elms (*N* = 7). (B) Mobility (√mm/s) of *S. punctillum* and *C. rufilabris* was significantly reduced after 3.5 h of exposure to spider mites reared on foliage from imidacloprid-treated elms compared to untreated elms. Means±s.e.m. marked with asterisks are significantly different at *P*<0.05.

Two important taxa of spider mite predators, Coccinellidae in the genus *Stethorus* and Chrysopidae were collected at both locations in most years of the study. Several previous studies indicate that lady beetles in the genus *Stethorus* are highly specialized predators of spider mites and are known to exhibit positive density dependence with increasing prey populations [44]–[46]. Green lacewing larvae, Chrysopidae, are also important generalist predators of spider mites [44], [45]. It was not surprising that *Stethorus* and Chrysopidae were rare on untreated trees in New York and Maryland, where populations of spider mites remained relatively low in all years of the study (Figures 1 and S1). However, the lack of numerical response by either predator to eruptive mite populations at both sites in all years was perplexing and unexpected. This observation and the fact that others have documented debilitating effects of imidacloprid on predatory insects [35], [36], [41], [42] led us to examine the effects of imidacloprid on the behaviour and longevity of two model predators of spider mites, *Stethorus punctillum* and *Chrysoperla rufilabris.* We found that feeding rates of adult *S. punctillum* and larval *C. rufilabris* were significantly reduced when *T. schoenei* from elms treated with imidacloprid were offered as prey for 3.5 h (*S. punctillum*, *F*_1,12_ = 56.62, *P*<0.001; *C. rufilabris*, F_1,12_ = 44.09, *P*<0.001; Figure 2A). While there were significant time by treatment interactions for both predators (*S. punctillum, F*_3,36_ = 34.63, *P<*0.01; *C. rufilabris, F*_3,36_ = 17.79, *P<*0.01), feeding rates differed significantly between treatments after only 1.5 h and differences grew larger as the experiment progressed (Table S5).

Consumption of prey tainted with imidacloprid directly affected mobility of predators thereby contributing to reduced feeding rates. *S. punctillum* and *C. rufilabris* were rapidly intoxicated when exposed to *T. schoenei* that consumed foliage from trees treated with imidacloprid. Their mobility was reduced after 3.5 h of exposure to *T. schoenei* from imidacloprid-treated elms (*S. punctillum*, *F*_1,12_ = 30.95, *P*<0.001; *C. rufilabris*, *F*_1,12_ = 32.08, *P*<0.001; Figure 2B), and the effect was evident after 0.5 h of exposure (Table S6). To determine if the route of exposure was through prey or through contact with imidacloprid-treated foliage, *S. punctillum* and *C. rufilabris* were maintained on leaves without *T. schoenei*. When predators were exposed to leaves from treated or untreated plants, no differences in mobility were observed (*S. punctillum*, *F*_1, 84.9_ = 0.70, *P* = 0.4; *C. rufilabris*, *F*_1,86.4_ = 0.18, *P* = 0.67; Table S6). Time did not interact with treatment with respect to mobility of the predators (*S. punctillum*, *F*_9,61_ = 0.5, *P*<0.87; *C. rufilabris*, *F*_9,61_ = 1.12, *P*<0.36). Exposure to imidacloprid or its metabolites appears to occur by ingesting contaminated prey rather than via cuticular absorption from contaminated leaf surfaces. In addition to impaired mobility, predators exhibited clear signs of intoxication including partial or complete lack of response to touch, tremors, regurgitation, excessive grooming, and inability to right themselves when placed on their back.

Consuming prey from leaves of imidacloprid-treated plants dramatically reduced longevity of both predators (*S. punctillum*, *t* = 23.04, df = 10.6, *P*<0.01; *C. rufilabris*, *t* = 4.66, df = 6.1, *P*<0.01). *S. punctillum* that consumed mites from untreated trees lived 9.34±0.30 (s.e.m.) out of a 10-day observation period, while those that consumed prey from treated plants lived less than one day on average, 0.89±0.21 (s.e.m.). *C. rufilabris* lived 12.64±2.18 (s.e.m.) days out of a possible 20 days after consuming mites from untreated plants compared to 2.13±0.18 (s.e.m.) days when offered *T. schoenei* from treated plants.

### Mechanisms underlying outbreaks of *T. schoenei*: Stimulatory effects of imidacloprid on spider mite reproduction

**Figure 3:**
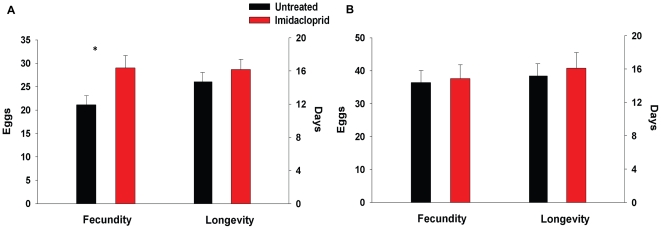
Effect of exposure to imidacloprid on lifetime fecundity and longevity of *T. schoenei.* (A) Effect of imidacloprid delivered through plant tissue. *T. schoenei* consuming foliage from elms treated with imidacloprid (*N* = 8) laid significantly more eggs than spider mites feeding on leaves from untreated trees (*N* = 8), while their longevity was not affected. (B) Topical application of imidacloprid to *T. schoenei.* Females (*N* = 30) sprayed with imidacloprid did not exhibit higher fecundity or longevity than *T. schoenei* sprayed with water (*N* = 30). Means±s.e.m. marked with asterisks are significantly different at *P*<0.05.

**Figure 4:**
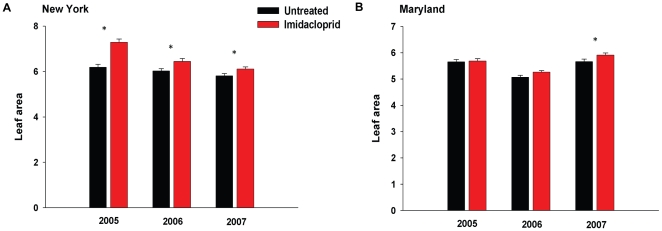
Effect of imidacloprid on elm leaf area in New York (A) and Maryland (B). Figure depicts area of leaves (√cm^2^) used in surveys of arthropod community presented in Figure 1. Elms (treated: *N* = 10; untreated: *N* = 10) in New York had a significantly greater leaf area following exposure to imidacloprid in all sampling years, while trees in Maryland (treated: *N* = 10; untreated: *N* = 10) were larger in the last year of the study. Means±s.e.m. marked with asterisks are significantly different at *P*<0.05.

Imidacloprid clearly affected reproduction of *T. schoenei* thereby revealing another deleterious consequence of its use. In microcosms containing leaves, imidacloprid directly enhanced fecundity of *T. schoenei*. Adult *T. schoenei* fed foliage from elms treated with imidacloprid laid more eggs than *T. schoenei* that consumed leaves from untreated elms (*F*_1,15_ = 4.93, *P* = 0.042; Figure 3A). While fecundity was enhanced by almost 40%, longevity was not affected (*F*_1,15_ = 1.54, *P* = 0.23; Figure 3A). Conversely, a direct stimulatory effect of imidacloprid on *T. schoenei* fecundity was absent when mites were directly sprayed with the pesticide and then offered foliage from elms free of imidacloprid (*F*_1,58_ = 0.52, *P* = 0.49; Figure 3B). The longevity of *T. schoenei* was similarly unaffected by dermal exposure to imidacloprid (*F*_1,58_ = 1.45, *P* = 0.23; Figure 3B). Females sprayed directly with imidacloprid may have been exposed to lower doses of the pesticide than *T. schoenei* feeding on imidacloprid-treated foliage for extended period of time, thus explaining lack of effect of dermal sprays on *T. schoenei* fecundity and longevity. Stimulation of *T. schoenei's* reproductive performance, however, could also be mediated through a physiological response of elms to imidacloprid. Evidence for altered plant physiology was evident in comparisons of leaf areas of treated and untreated elms. Despite housing greater numbers of spider mites, imidacloprid-treated trees had significantly larger leaves in New York each year (2005, *F*_1,89_ = 17.89, *P*<0.001; 2006, *F*_1,54_ = 6.83, *P* = 0.009; 2007, *F*_1,72_ = 4.57, *P* = 0.033), and in 2007 in Maryland (*F*_1,54_ = 5.54, *P = *0.02; Figure 4). The interaction between time and treatment did not have significant effects on leaf sizes (New York: 2005, *F*_4,89_ = 0.15, *P* = 0.963; 2006, *F*_2,54_ = 0.09, *P* = 0.915; 2007, *F*_3,72_ = 0.13, *P* = 0.944; Maryland: 2007, *F*_2,54_ = 1.2, *P = *0.303). Contrary to an earlier report [14], we found that increased leaf size was not accompanied by increased nitrogen content (Table S7).

## Discussion

Applications of imidacloprid significantly altered the community of arboreal arthropods associated with elms in Central Park, New York and College Park, Maryland. Spider mites responded strongly to applications of imidacloprid and were far more abundant on treated elms (Figure 1, Table S2). These findings accord with others were pesticides other than imidacloprid reduced the richness, biomass, diversity or abundance of arthropods in apple orchards [17], tomato farms [19], oat fields [20] and corn fields [18]. One striking similarity between this investigation and studies in agricultural systems was the increased abundance of mites following application of pesticides [17], [18]. Several of these studies noted significant reductions in the abundance of many predatory taxa following these applications [18]–[20].

Although previous studies did not examine changes in community structure, elevated populations of spider mites following applications of imidacloprid have been documented previously in systems involving *Gleditsia triacanthos* (honeylocust) and *Platytetranychus multidigituli* (honeylocust spider mite) [15], *Tsuga canadensis* (Canadian hemlock) and *Oligonychus ununguis* (spruce spider mite) [9] and *Rosa* sp. (rose) and *Tetranychus urticae* (twospotted spider mite) [14]. It is noteworthy that elevated populations of mites occurred in several distinct plant families and the possibility of common mechanisms is intriguing. Debilitation of key natural enemies has been suggested as a likely cause of mite outbreaks [15], and our field studies suggest that a loss of top-down suppression of *T. schoenei* played a role in spider mite outbreaks observed in New York and Maryland. We found evidence that imidacloprid removed the most abundant natural enemy of spider mites in our study, *Galendromus* spp.. The abundance of *Galendromus* spp. was lower on imidacloprid-treated trees in 2007 in New York and in 2006 and 2007 in Maryland, while the predatory mites did not differ in their abundance between imidacloprid-treated and untreated elms in the remaining years of the study (Table S2). The fact that imidacloprid did not consistently remove *Galendromus* spp. from imidacloprid-treated elms in each year suggests that other factors played a role in population dynamics of this predator. While the response of *Galendromus* spp. to imidacloprid exposure in the field has not been examined before, several laboratory [38], [39] and greenhouse [37] studies report toxicity of imidacloprid to *Galendromus* spp.. Moreover, in a laboratory experiment, imidacloprid exposure had a negative impact on functional responses of phytoseiid mites from other genera (*Neoseiulus* and *Phytoseiulus*) [40], and it is possible that foraging of *Galendromus* spp. was affected by imidacloprid in the present study as well. Further research is necessary to establish if imidacloprid exposure dampens foraging efficiency and lowers survival of *Galendromus* spp. in the field.

Whereas the phytoseiid predators were likely able to persist on untreated elms owing to abundant alternative prey, the eriophyid mites, untreated trees had few arthropods that could serve as prey to *Stethorus* and Chrysopidae. Thus, lack of the key insect predators on these trees is not surprising. Their absence on imidacloprid-treated trees, however, suggests that imidacloprid had a negative effect on the insect predators of spider mites. Laboratory assays clearly demonstrated debilitating consequences of imidacloprid exposure to two model insect predators of spider mites, *S. punctillum* and *C. rufilabris.* Consumption of *T. schoenei* fed leaves from imidacloprid-treated elms reduced feeding and mobility and shortened longevity of both predators. Our results concur with others that show lethal and sublethal effects of imidacloprid exposure to lady beetles, lacewings, and parasitic wasps [36], [41], [42], [47]. It is clear from our laboratory experiments that *S. punctillum* and *C. rufilabris* gained exposure to imidacloprid through consumption of tainted prey and were adversely affected. Prey-mediated toxicity of imidacloprid to predators such as Coccinellidae and Chrysopidae, may have precluded successful colonization and positive density dependence of important insect predators on elms treated with imidacloprid.

Enhanced fecundity of *T. schoenei* on elms treated with imidacloprid likely contributed in a significant way to eruptive populations of spider mites in New York and Maryland. Elevated fecundity of *T. schoenei* in this report is consistent with studies of imidacloprid's effects on a related spider mite, *T. urticae,* in some [31], [32], but not other studies [48]. Enhanced fecundity can play an important contributory role in outbreaks especially for multivoltine pests like spider mites [29], [49]. Dramatic increases in *T. schoenei* abundance late in the growing season are consistent with previously documented outbreaks of spider mites in urban settings related to elevated temperatures, pollution, or loss of top-down suppression by natural enemies [49], [50].

Notably, this is the first study to show that imidacloprid enhances spider mite fecundity via a plant-mediated mechanism. We demonstrated that imidacloprid exposure enhances reproduction of spider mites only when spider mites feed on foliage from imidacloprid-treated elms. This bottom-up effect of the insecticide implies altered quality of the plant for spider mites. Other studies tie imidacloprid's influence on plant physiology to pest outbreaks as well. Increased fecundity and resurgence of a lepidopteran herbivore, pyralid moth *Tryporyza incertulas*, was linked to decreased activity of a detoxification enzyme, glutathione S-transferase in rice treated with imidacloprid [51], [52]. Moreover, *Rosa* sp. treated with imidacloprid housed greater numbers of spider mites and had significantly greater chlorophyll indices, leaf area, and nitrogen concentration than untreated plants [14]. We suggest that imidacloprid alters plants in a way that enhances the quality of plants as hosts for *T. schoenei* and consistently larger elm leaves lend support to this hypothesis. Further, imidacloprid was recently shown to induce expression of genes involved in plant defenses against pathogens [53] and reduce expression of protease inhibitor genes employed in plant defense against herbivores [54]. It is noteworthy that separate pathways regulate plant defenses against pathogens and herbivores, and in several model plants, the two pathways exhibit antagonistic interactions [55]. If imidacloprid applications result in deployment of pathogen-related responses, pivotal plant defenses against herbivores could be adversely affected. Decreased expression of genes for protease inhibitors, for example, which reduce digestibility of plant proteins, would allow spider mites to assimilate nutrients more efficiently and may explain their increased fecundity on imidacloprid-treated plants. Certainly, the mechanisms by which imidacloprid alters plant physiology and leads to enhanced herbivore performance and ultimately changes the entire arthropod community deserve further examination.

Previous accounts attributed secondary outbreaks of pests coincident with area-wide eradication programs such as the one is Central Park to disruption of top-down regulation by predatory arthropods [26], [29], [56]. Our study indicates that plant-mediated enhancement of mite reproduction may conspire with relaxation of suppression by predators to elevate mite populations on trees treated with imidacloprid. A holistic approach that considers both top-down and bottom-up forces will lead to a clearer understanding of the mechanisms underlying pest outbreaks following the application of pesticides [27], [29]. This is especially important in light of increasing pressure to mitigate deleterious ecological and economic impacts linked to the ongoing global deluge of invasive species.

## Materials and Methods

### Applications of imidacloprid

In Central Park, applications of imidacloprid were administered in the spring of each year, and doses of the pesticide were calibrated based on trunk diameter of each *U. americana* measured at a standardized height of approximately 150 cm from the ground. This is a standard procedure for determining appropriate doses of pesticides applied to trees. Certified pesticide applicators used two formulations and three methods of application for imidacloprid. All methods and doses of pesticides were in compliance with manufacturers' requirements and recommendations mandated by APHIS, the federal agency imposing the treatments. In 2005, 1323 trees received trunk injections of liquid imidacloprid formulated as 10% active ingredient (Imicide® Hp, J.J. Mauget Co., Arcadia, CA, USA) applied at the rates of 2 ml for every 2.54 cm of trunk diameter for trees ranging between 5.1 to 27.9 cm of trunk diameter, 4 ml per 2.54 cm for trees with trunk diameters of 30.5 to 58.42 cm, 8 ml per 2.54 cm for trees 61.0 to 88.9 cm of trunk diameter, and 12 ml per 2.54 cm for trees greater than 88.9 cm trunk diameter. Twenty eight trees received a soil drench of imidacloprid formulated as a 75% active ingredient wettable soluble powder and 3483 trees received a soil injection of Merit® 75 WSP or Bandit® 75 WSP (Bayer Environmental Science, Research Triangle Park, NC, USA) at the rate of 227 g per 2.54 cm of trunk diameter. In 2006, 1448 trees received trunk injections of imidacloprid (Imicide® Hp), 12 trees received soil drenches of imidacloprid (Merit® 75 WSP) at the rate of 113 g per 2.54 cm of trunk diameter, and 3401 received imidacloprid as soil injections (Merit® 75 WSP or Bandit® 75 WSP) at the rate of 113 g per 2.54 cm of trunk diameter. In 2007, 301 trees received trunk injections of imidacloprid (Imicide® Hp) and 4716 received imidacloprid as soil injections (Merit® 75 WSP or Bandit® 75 WSP) at the rate of 113 g per 2.54 cm of trunk diameter. Table S8 details the number of trees in different sizes classes treated by different methods of application for all years of the study. All trees outdoors and in the greenhouse at College Park received imidacloprid formulated as a 75% active ingredient wettable powder (Merit® 75 WP) at the rate of 1.96 g per 2.54 cm of trunk diameter applied by A.S., S.F.C., and M.J.R. All methods of imidacloprid applications used in New York and Maryland are commonly employed to suppress susceptible insect herbivores on trees in urban and residential landscapes. Imidacloprid at both locations was applied at label dose specified by the manufacturer of each pesticide formulation.

### Community structure in New York and Maryland

Central Park was in the federal quarantine zone established by USDA-APHIS to eradicate *A. glabripennis* [57]. The agency imposed mandatory applications of pesticides to trees within an 800-m radius of every confirmed *A. glabripennis* infestation [57]. Thus, while each tree was treated independently, there was a spatial limitation to the random assignment of trees to treatments. We recognize that this limitation restricts inferences only to trees sampled in Central Park, hence, the need for a second study site in Maryland. In Central Park, 86^th^ Street marked the boundary for the eradication zone, and all elms located south of 86^th^ Street received individual treatments of imidacloprid. Each year, ten different treated and untreated elms were sampled. All trees were mature, and measured approximately 10–30 m in height. In 2005, trees on two east-west transects across the park north and south of 86^th^ Street were sampled in untreated and treated zones, respectively. In 2006 trees were sampled on the western boundary of the park parallel to 8^th^ Avenue north and south of the treatment demarcation line at 86^th^ Street and in 2007, trees along the eastern boundary along 5^th^ Avenue north and south of the demarcation boundary were included in the study. Elms were sampled every three to six weeks and leaf samples were collected five times in 2005, three times in 2006 and four times in 2007. The experiment was repeated in Maryland using a stand of *U. americana* in a maintained margin lining a boulevard. In Maryland, 20 *U. americana* were randomly assigned to two treatments. Half received annual applications of imidacloprid each spring and half served as untreated controls. *U. americana* in the Maryland study were younger than those in New York and measured approximately 3–5 m in height. Ten treated and ten untreated elms were sampled every two to six weeks for three consecutive years.

In all years at both locations, four branches approximately 15–30 cm long were removed from each cardinal position on each tree. Due to the height of the canopies of trees in New York, pole pruners were used in combination with hand pruners to collect samples. Small trees in Maryland with lower canopies were sampled with hand pruners. The excised foliage from each tree was collectively bagged, placed in a cooler, and brought back to the laboratory and refrigerated until arthropods were counted using a dissecting microscope. This method of sampling has been used to estimate densities of mites and their predators in a wide variety of trees in landscapes [15], [58]–[60] and agricultural fields [61]–[63]. All arthropods on the adaxial and abaxial surfaces of the two most terminal leaves were counted, and natural enemies and their eggs were noted on three additional leaves occupying position 3–5 on the branch's terminus. Abundance of all arthropods was tallied as a function of measured leaf area (cm^2^) recorded for each leaf using an area meter (LI-COR Environmental, Lincoln, NE, USA). In 2005, all leaves collected at both sites were dried at 70°C for seven days and ground in a plant mill (Capitol Scientific, Austin, USA). Percent nitrogen content of the samples was analyzed by the Environmental Analysis Research Lab at the University of Maryland using dry combustion method [64].

### Mechanisms underlying outbreaks of *T. schoenei*: Deleterious effects of imidacloprid on two model insect predators

In a greenhouse, 14 *U. americana* planted in containers were randomly assigned to one of two treatments. Half received imidacloprid applications as described above for the Maryland study and half were assigned as untreated controls. Trees were arranged in a randomized complete block design in two rows within the greenhouse space. Trees were 1.8 m apart and canopies of individual trees were not in contact with adjacent elms. After imidacloprid applications, all trees received branches infested with *T. schoenei* from an untreated elm to establish colonies of mites. *T. schoenei* were allowed to multiply for two months prior to bioassays. To assess insecticide-related changes in foraging and mobility of the predators we followed the protocol of James and Price [31] previously used to measure spider mite fecundity. Leaves were removed from treated and untreated trees and leaf disks 22 mm in diameter were punched from each leaf with an apple corer (Progressive International, Kent, WA, USA). Leaf disks were cleaned of mites and placed lower side down in a 40 mm Petri dish filled with saturated cotton gauze. Ten adult female mites were transferred from each tree and placed on the respective leaf disk. Commercially purchased (IPM Labs, Locke, NY, USA) predators, a single adult *S. punctillum* or a larva of *C. rufilabris* was then introduced to the leaf disk. The number of mites consumed was recorded after 0.5, 1.5, 2.5, and 3.5 h of exposure. At each time-interval, predators were removed from arenas to measure their mobility. Mobility was assessed by placing a predator at the center of a 40 mm diameter circle, and recording the time required to escape from the circle.

In a separate experiment, predators were also exposed to leaves from treated and untreated trees without prey to determine if dermal exposure to contaminated leaf surfaces affected mobility thereby separating the effect of intoxication through contact with treated leaves from the effect of intoxication by ingestion of prey. An arena was constructed of a 118 mL Solo Cup (Solo Cup Company, Urbana, IL, USA). A water source was included which consisted of a trimmed micropipette tip stuffed with water-saturated cotton gauze. Each arena was supplied with 2–3 excised leaves from treated or untreated elms from which all life stages of mites were removed. Mobility was assessed as in the previous assay at the same intervals of time. The bioassays for each predator species included at least three subsamples in all replicates and treatment combinations (*n* = 95 for *S. punctillum* and *n* = 84 for *C. rufilabris*).

The effect of consuming tainted prey on predator longevity was assessed by exposing *S. punctillum* adults and *C. rufilabris* larvae to the following treatments. *S. punctillum* adults or the *C. rufilabris* larvae were individually placed in a 118 mL Solo Cup and supplied with a trimmed micropipette tip filled with cotton saturated with a sugar-water solution (10 mg sugar: 100 mg water). Leaves infested with *T. schoenei* from treated or untreated elms described in the previous study were provided at the beginning of the experiment and replaced every other day thereafter to ensure that prey were not limiting. The arenas were held under lighted ambient laboratory conditions (23±2°C) for the duration of the assays. Every 24 hours, predators were observed and considered dead if they were completely unresponsive to the touch of a probe and not making any movements. Assays with *C. rufilabris* were conducted for 20 days and assays for *S. punctillum* were conducted for 10 days. Trees were replicates and predators were subsamples (2 or 3 predators per tree × 7 trees).

### Mechanisms underlying outbreaks of *T. schoenei*: Stimulatory effects of imidacloprid on spider mite reproduction

In a common garden experiment, an additional 16 *U. americana* were planted at the University of Maryland Turf Research Farm at College Park, Maryland in May 2005. The trees were uniform in size and measured 1.5 m at the time of planting. The elms were randomly assigned to one of two treatments. Half received imidacloprid and half of the trees were untreated controls. Imidacloprid was applied in June 2006 and May 2007. Foliage from these elms was used in all experiments assessing *T. schoenei* fecundity, which were carried out in August and September 2007. One month before bioassays with *T. schoenei* were initiated, *T. schoenei* colonies were established in the laboratory from naturally occurring populations and maintained on leaves from untreated elms in growth chambers (Percival Scientific, Perry, IA, USA) at 23±2°C and 16∶8 light∶ dark. *T. schoenei* were then removed from colonies of mites reared on insecticide-free trees for at least two generations and used in the assays. One hundred and forty four *T. schoenei* females of the same age were randomly assigned to treated or untreated leaves and enclosed in 60 mm plastic clip cages. Mites were transferred to new leaves every other day. Leaves with mites in clip cages were placed in growth chambers maintained at 23±2°C and 16∶8 light∶ dark. Lifetime fecundity and longevity were recorded for all females maintained on foliage from eight treated and eight untreated elms. Trees were replicates and individual *T. schoenei* were subsamples (nine per replicate).

To test effects of direct exposure to imidacloprid, 60 even-aged females reared on foliage from insecticide-free trees were randomly assigned to one of two treatments. Half of the females received sprays of imidacloprid and half received sprays of distilled water delivered by a Potter Spray Tower® (Burkard, Rickmansworth, UK). Two mL of flowable formulation of Admire® (2 g of imidacloprid/L, Bayer Environmental Science) were delivered at 50 kPa, resulting in an average application of 1.6–1.8 mg of liquid per cm^2^. Imidacloprid applied at this rate to bean leaves was previously shown to enhance spider mite fecundity [31]. Females were enclosed in clip cages and maintained on insecticide-free leaves for the duration of the experiment in growth chambers under conditions described previously. Lifetime fecundity and longevity were measured. In this experiment, individual females were replicates.

### Statistical analyses

To test and visualize how the community of arthropods responded to imidacloprid treatment through time, we utilized a constrained form of principal components analysis called principal response curve (PRC), a multivariate approach based on redundancy analysis [18], [65], [66]. It performs weighted least-squares regression of values of inert and latent variables, referred to as axes, extracted from the species abundance data on treatment and time. The weights are based on abundance of each taxon relative to its accumulation in the control treatment; therefore, response of the sampled arthropod fauna is expressed as deviation from the community in control treatment. The analysis provides an exact significance test. Monte-Carlo permutations are used to test for significance of the response curve. An *F* test statistic is calculated and the permutations produce 1,000 new data sets that are equally likely under a null hypothesis of canonical coefficients equalled zero. Significance is then computed based on the proportion of *F* values greater or equal to the *F* value of the original data set [66]. In addition to examining community structure with canonical coefficients, PRC scores were used to examine responses of individual taxa to insecticide applications. Following the convention of Dively [18], taxa with coefficients near zero (0.5 to −0.5) were considered to have no response or one unrelated to the overall pattern expressed by the PRCs.

Effects of imidacloprid treatments on spider mite densities, prey consumption, predator mobility, predator longevity, and mite fecundity and longevity were evaluated by analysis of variance with repeated measures, randomized complete block analysis of variance, or two sample t-tests. Transformations corrected heteroschedastic data prior to analyses. Non-parametric Kruskal-Wallis tests (χ*^2^* statistic) were used when assumptions of parametric analysis could not be satisfied [67].

## Supporting Information

Figure S1 Abundance (√number/cm^2^) of the spider mite, *T. schoenei,* on elms treated with imidacloprid (*N* = 10) and on untreated trees (*N* = 10) in New York (A) and Maryland (B). Asterisks mark means±s.e.m. that differed significantly within each sampling date (*P*<0.05) (Tukey's test).
(TIF)

Table S1 Comparisons of abundance of *T. schoenei* on elms treated with imidacloprid and untreated elms in New York (NY), and Maryland (MD).
(DOC)

Table S2 Comparison of abundance of Tydeidae, Diptilomiopidae and Phytoseiidae on elms treated with imidacloprid and untreated trees in New York (NY) and Maryland (MD).
(DOC)

Table S3 Comparisons of abundance (number/cm^2^) of Eriococcidae on elms treated with imidacloprid and untreated elms in Maryland.
(DOC)

Table S4 Species scores were generated by PRC analysis to examine responses of individual taxa to imidacloprid applications.
(DOC)

Table S5 Comparison of feeding rates of *S. punctillum* and *C. rufilabris* exposed to spider mites that consumed foliage from imidacloprid-treated elms and untreated elms.
(DOC)

Table S6 Comparison of mobility of *S. punctillum* and *C. rufilabris* exposed to imidacloprid in prey and foliage.
(DOC)

Table S7 Comparison of nitrogen levels in elm trees treated with imidacloprid in New York (NY) and Maryland (MD) in 2005
(DOC)

Table S8 The number of trees in different size classes treated by different methods of application for 2005, 2006, and 2007.
(DOC)

## References

[1] Walther GR, Roques A, Hulme PE, Sykes MT, Pysek P (2009). Alien species in a warmer world: risks and opportunities.. Trends in Ecology & Evolution.

[2] Corn ML, Buck EH, Rawson J, Segarra A, Fischer E (2002). Invasive non-native species: Background and issues for Congress. RL30123.. Congressional Research Service.

[3] Pimentel D, Morrison D, Zuniga R (2005). Update on the environmental and economic costs associated with alien-invasive species in the United States.. Ecological Economics.

[4] Kovacs KF, Haight RG, McCullough DG, Mercader RJ, Seeger NW (2009). Cost of potential emerald ash borer damage in U.S. communities, 2009–2019.. Ecological Economics.

[5] Raupp MJ, Buckelew Cumming A, Raupp EC (2006). Street tree diversity in eastern North America and its potential for tree loss to exotic pests.. Journal of Arboriculture.

[6] Raupp MJ, Szczepaniec A, Buckelew Cumming A (2007). Prophylactic pesticide applications and low species diversity: Do they create pest outbreaks in the urban forest?. Interagency Research on Invasive Species [USDA Proc forum].

[7] Cappaert D, McCullough DC, Poland TM, Siegert NW (2005). Emerald ash borer in North America: A research and regulatory challenge.. American Entomologist.

[8] Mullins JW (1993). Imidacloprid: a new nitroguanidine insecticide.. American Chemical Society.

[9] Raupp MJ, Webb R, Szczepaniec A, Booth D, Ahern R (2004). Incidence, abundance, and severity of mites on hemlocks following applications of imidacloprid.. Journal of Arboriculture.

[10] Frank S, Ahern R, Raupp MJ (2007). Does imidacloprid reduce defoliation by Japanese beetles on linden for more than one growing season?. Journal of Arboriculture.

[11] Szczepaniec A, Raupp MJ (2007). Residual toxicity of imidacloprid to hawthorn lace bug, *Corythuca cydoniae*, feeding on cotoneasters in landscapes and containers.. Journal of Environmental Horticulture.

[12] Cowles RS, Montgomery ME, Cheah CSJ (2006). Activity and residues of imidacloprid applied to soil and tree trunks to control hemlock woolly adelgid (Hemiptera: Adelgidae) in forests.. Journal of Economic Entomology.

[13] Tomizawa M, Casida JE (2003). Selective toxicity of neonicotinoids attributable to specificity of insect and mammalian nicotinic receptors.. Annual Review of Entomology.

[14] Gupta G, Krischik VA (2007). Professional and consumer insecticides for the management of adult Japanese beetle on hybrid tea rose.. Journal of Economic Entomology.

[15] Sclar DC, Gerace D, Cranshaw WS (1998). Observations of population increase and injury by spider mites (Acari: Tetranychidae) on ornamental plants treated with imidacloprid.. Journal of Economic Entomology.

[16] Berliner W (2008). Associate Vice-President of Horticulture, Central Park Conservancy, Personal communication..

[17] Brown MW, Adler CRL (1989). Community structure of phytophagous arthropods on apple.. Environmental Entomology.

[18] Dively GP (2005). Impact of transgenic VIP3A x Cry1Ab lepidopteran-resistant field corn on the nontarget arthropod community.. Environmental Entomology.

[19] Letourneau DK, Goldstein B (2001). Pest damage and arthropod community structure in organic vs. conventional tomato production in California.. Journal of Applied Ecology.

[20] Suttman CE, Barrett GW (1979). Effects of sevin on arthropods in an agricultural and old-field plant community.. Ecology.

[21] Berenzen N, Kumke T, Schulz HK, Schulz R (2005). Macroinvertebrate community structure in agricultural streams: impact of runoff-related pesticide contamination.. Ecotoxicology and Environmental Safety.

[22] Schafer RB, Caquet T, Siimes K, Mueller R, Lagadic L (2007). Effects of pesticides on community structure and ecosystem functions in agricultural streams of three biogeographical regions in Europe.. Science of the Total Environment.

[23] Fairchild WL, Eidt DC (1993). Perturbation of the aquatic invertebrate community of acidic bog ponds by the insecticide fenitrothion.. Archives of Environmental Contamination and Toxicology.

[24] Rohr JR, Crumrine PW (2005). Effects of an herbicide and an insecticide on pond community structure and process.. Ecological Applications.

[25] Luck RF, Dahlsten DL (1975). Natural decline of a pine needle scale (*Chionaspis pinifoliae* (Fitch)) outbreak at South Lake Tahoe, California, following cessation of adult mosquito control with malathion.. Ecology.

[26] Dreistadt SH, Dahlsten DL (1986). Medfly eradication in California: lessons from the field.. Environment.

[27] Dutcher JD (2007). A review of resurgence and replacement causing pest outbreaks in IPM. In: Ciancio A, Mukerji KG, editors. General Concepts in Integrated Pest and Disease Management..

[28] Costamagna AS, Landis DA, Difonzo DC (2007). Suppression of soybean aphid by generalist predators results in a trophic cascade in soybeans.. Ecological Applications.

[29] Raupp MJ, Shrewsbury PM, Herms DH (2010). Ecology of herbivorous arthropods in urban landscapes.. Annual Review of Entomology.

[30] Luckey TD (1968). Insect hormoligosis.. Journal of Economic Entomology.

[31] James DG, Price TS (2002). Fecundity of twospotted spider mite (Acari: Tetranychidae) is increased by direct and systemic exposure to imidacloprid.. Journal of Economic Entomology.

[32] Chiriboga A (2009). Physiological responses of woody plants to imidacloprid formulations..

[33] Tenczar EG, Krischik VA (2006). Management of cottonwood leaf beetle (Coleoptera: Chrysomelidae) with a novel transplant soak and biorational insecticides to conserve coccinellid beetles.. Journal of Economic Entomology.

[34] Rebek EJ, Sadof CS (2003). Effects of pesticide applications on the euonymus scale (Homoptera: Diaspididae) and its parasitoid, *Encarsia citrina* (Hymenoptera: Aphelinidae).. Journal of Economic Entomology.

[35] Cole PG, Horne PA (2006). The impact of aphicide drenches on *Micromus tasmaniae* (Walker) (Neuroptera: Hemerobiidae) and the implications for pest management in lettuce crops.. Australian Journal of Entomology.

[36] Rogers MA, Krischik VA, Martin LA (2007). Effect of soil application of imidacloprid on survival of adult green lacewing, *Chrysoperla carnea*, (Neuroptera: Chrysopidae), used for biological control in greenhouse.. Biological Control.

[37] Stavrinides MC, Mills NJ (2009). Demographic effects of pesticides on biological control of Pacific spider mite (*Tetranychus pacificus*) by the western predatory mite (*Galendromus occidentalis*).. Biological Control.

[38] Bostanian NJ, Thistlewood HA, Hardman JM, Laurin MC, Racette G (2009). Effect of seven new orchard pesticides on *Galendromus occidentalis* in laboratory studies.. Pest Management Science.

[39] James DG (2003). Toxicity of imidacloprid to *Galendromus occidentalis*, *Neoseiulus fallacies*, *Amblyseius andersoni* (Acari: Phytoseiidae) from hops in Washington State, USA.. Experimental and Applied Acarology.

[40] Poletti M, Maia AHN, Omoto C (2007). Toxicity of neonicotinoid insecticides to *Neoseiulus californicus* and *Phytoseiulus macropilis* (Acari: Phytoseiidae) and their impact on functional response to *Tetranychus urticae* (Acari: Tetranychidae).. Biological Control.

[41] James DG (2003). Pesticide susceptibility of two coccinellids (*Stethorus punctum picipes* and *Harmonia axyridis*) important in biological control of mites and aphids in Washington hops.. Biocontrol Science and Technology.

[42] Papachristos DP, Milonas PG (2008). Adverse effects of soil applied insecticides on the predatory coccinellid *Hippodamia undecimnotata* (Coleoptera: Coccinellidae).. Biological Control.

[43] Mooney HA, Cleland EE (2001). The evolutionary impact of invasive species.. Proceedings of National Academy of Sciences.

[44] Chazeau J, Helle W, Sabelis MW (1985). in Spider Mites.. Their Biology, Natural Enemies and Control.

[45] McMurtry MA, Huffaker CB, Van de Vrie M (1970). Ecology of tetranychid mites and their natural enemies: A review. In: Tetranychid mites: their biological characteristics and the impact of spray practices.. Hilgardia.

[46] Hull LA, Asquith D, Mowery PD (1976). Distribution of *Stethorus punctum* in relation to densities of the European red mite.. Environmental Entomology.

[47] Rogers ME, Potter DA (2003). Effects of spring imidacloprid application for white grub control on parasitism of Japanese beetle (Coleoptera: Scarabaeidae) by *Tiphia vernalis* (Hymenoptera: Tiphiidae).. Journal of Economic Entomology.

[48] Ako M, Poehling HM, Borgemeister C, Nauen R (2006). Effect of imidacloprid on the reproduction of acaricide-resistant and susceptible strains of *Tetranychus urticae* Koch (Acari: Tetranychidae).. Pest Management Science.

[49] Kropczynska D, van de Vrie M, Tomczyk A (1988). Bionomics of *Eotetranychus tiliarium* and its phytoseiid predators.. Experimental and Applied Acarology.

[50] Schneider K, Balder H, Jackel B, Pradel B, Backhaus GF, Balder H, Idczak E (2000). Bionomics of *Eotatranychus tiliarum* as influenced by key factors.. International Symposium on Plant Health in Urban Horticulture.

[51] Wu JC (2003). Impacts of pesticides on physiology and biochemistry of rice.. Scientia Agriculatura Sinica.

[52] Wang AH, Wu JC, Yu YS, Liu JL, Yue JF (2005). Selective insecticide-induced stimulation on fecundity and biochemical changes in *Tryporyza incertulas* (Lepidoptera: Pyralidae).. Journal of Economical Entomology.

[53] Ford KA, Casida JE, Chandran D, Gulevich AG, Okrent RA (2010). Neonicotinoid insecticides induce salicylate-associated plant defense responses.. Proceedings of National Academy of Science.

[54] Szczepaniec A (2009). Mechanisms underlying outbreaks of spider mites following applications of imidacloprid. PhD thesis..

[55] Doares SH, Narvaez-Vasquez J, Conconi A, Ryan CA (1995). Salicylic acid inhibits synthesis of proteinase inhibitor in tomato leaves induced by systemin and jasmonic acid.. Plant physiology.

[56] DeBach P, Rose M (1977). Environmental upsets caused by chemical eradication.. California Agriculture.

[57] USDA APHIS (2007). Asian Longhorned Beetle Cooperative Eradication Program in the New York Metropolitan Area..

[58] Kropczynska D, Czajkowska B, Tomczyk A, Kielkiewicz M, Bernini F, Nannelli R, Nuzaci G, DeLillio E (2002). Mite communities on linden trees (*Tilia* sp.) in an urban environment.. In Acarid phylogeny and evolution: Adaptation in mites and ticks.

[59] Balder H, Jackel B, Pradel B (1999). Investigations on the existence of beneficial organisms on urban trees in Berlin.. Acta Horticulturae.

[60] Shrewsbury P, Hardin M (2003). Evaluation of predatory mite (Acari: Phytoseiidae) releases to suppress spruce spider mites, *Oligonychus ununguis* (Acari: Tetranychidae), on juniper.. Journal of Economic Entomology.

[61] Le Roy M, Brodeur J, Cloutier C (1999). Seasonal abundance of spider mites and their predators on red raspberry in Quebec, Canada.. Environmental Entomology.

[62] Pratt P, Croft B (2000). Screening of predatory mites as potential control agent of pest mites in landscape plant nurseries of the Pacific Northwest.. Journal of Environmental Horticulture.

[63] Croft B, Slone D (1997). Equilibrium densities of European red mite (Acari: Tetranychidae) after exposure to three levels of predacious mite diversity on apple.. Environmental Entomology.

[64] Yeomans JC, Bremner JM (1991). Carbon and nitrogen analysis of soils by automated combustion techniques.. Communications in Soil and Plant Analysis.

[65] van den Brink PJ, ter Braak CJF (1999). Principal response curves: analysis of time-dependent multivariate responses of a biological community to stress.. Environmental Toxicology and Chemistry.

[66] Prasifka JR, Hellmich RL, Dively GP, Lewis LC (2005). Assessing the effects of pest management on nontarget arthropods: the influence of plot size and isolation.. Environmental Entomology.

[67] Zar J (1999). Biostatistical Analysis. 4^th^ ed..

