# Supplementary figures and images for: Neonicotinoid Insecticide Imidacloprid Causes Outbreaks of Spider Mites on Elm Trees in Urban Landscapes
Source: PLoS One. 2011 May 31;6(5):e20018. doi: 10.1371/journal.pone.0020018 (PMC3104998; doi:10.1371/journal.pone.0020018)

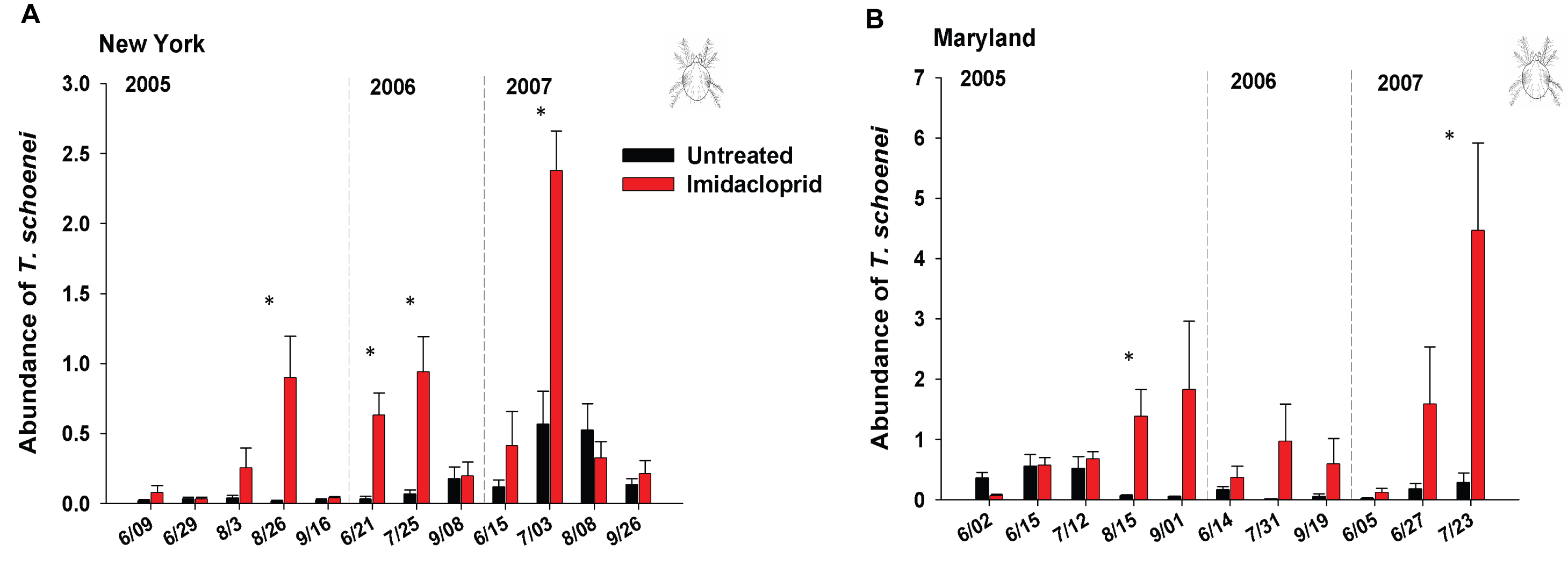

Supplement: Figure S1 — Abundance (√number/cm2) of the spider mite, T. schoenei, on elms treated with imidacloprid (N = 10) and on untreated trees (N = 10) in New York (A) and Maryland (B). Asterisks mark means±s.e.m. that differed significantly within each sampling date (P<0.05) (Tukey's test). (TIF) [file pone.0020018.s001.tif]
