# Supplementary material for: Neonicotinoid Insecticide Imidacloprid Causes Outbreaks of Spider Mites on Elm Trees in Urban Landscapes
Source: PLoS One. 2011 May 31;6(5):e20018. doi: 10.1371/journal.pone.0020018 (PMC3104998; doi:10.1371/journal.pone.0020018)
Supplement: Table S1 — Comparisons of abundance of T. schoenei on elms treated with imidacloprid and untreated elms in New York (NY), and Maryland (MD). (DOC) [file pone.0020018.s002.doc]

**Table S1**. Comparisons of abundance of *T. schoenei* on elms treated with imidacloprid and untreated elms in New York and Maryland.

| **New York** | | | | **Maryland** | | | |
| --- | --- | --- | --- | --- | --- | --- | --- |
| **Date** | **Test** | **df** | **P value** | **Date** | **Test** | **df** | **P value** |
| 6/09/05 | F = 0.95 | 1,18 | 0.347 | 6/02/05 | χ2 = 7.29 | 1 | 0.007 |
| 6/29/05 | χ2 = 0.05 | 1 | 0.819 | 6/15/05 | F = 1.06 | 1,18 | 0.317 |
| 8/03/05 | χ2 = 3.46 | 1 | 0.063 | 6/27/05 | F = 0.24 | 1,18 | 0.629 |
| 8/26/05 | F = 28.75 | 1,18 | <0.001 | 7/12/05 | F = 2.37 | 1,18 | 0.142 |
| 9/16/05 | χ2 = 0.01 | 1 | 0.940 | 8/15/05 | F = 18.73 | 1,18 | 0.001 |
| 6/21/06 | F = 2.38 | 1,18 | 0.140 | 9/01/05 | F = 14.10 | 1,18 | 0.002 |
| 7/25/06 | χ2 = 4.08 | 1 | 0.001 | 6/14/06 | χ2 = 0.08 | 1 | 0.775 |
| 9/08/06 | χ2 = 6.90 | 1 | 0.009 | 7/31/06 | χ2 = 7.06 | 1 | 0.008 |
| 6/15/07 | χ2 = 0.16 | 1 | 0.692 | 9/19/06 | F = 5.43 | 1,18 | 0.032 |
| 7/03/07 | χ2 = 0.03 | 1 | 0.869 | 6/05/07 | χ2 = 0.20 | 1 | 0.657 |
| 8/08/07 | χ2 = 11.07 | 1 | 0.001 | 6/27/07 | χ2 = 6.24 | 1 | 0.013 |
| 9/26/07 | F = 2.60 | 1,18 | 0.125 | 7/23/07 | F = 13.76 | 1,18 | 0.002 |

In all significant comparisons, *T. schoenei* abundance on treated trees exceeded that of untreated trees. Non-parametric Kruskal-Wallis tests (χ*^2^*) were used to compare *T. schoenei* abundance when assumptions of ANOVA could not be met through transformation of the data.
