# Supplementary material for: Neonicotinoid Insecticide Imidacloprid Causes Outbreaks of Spider Mites on Elm Trees in Urban Landscapes
Source: PLoS One. 2011 May 31;6(5):e20018. doi: 10.1371/journal.pone.0020018 (PMC3104998; doi:10.1371/journal.pone.0020018)
Supplement: Table S2 — Comparison of abundance of Tydeidae, Diptilomiopidae and Phytoseiidae on elms treated with imidacloprid and untreated trees in New York (NY) and Maryland (MD). (DOC) [file pone.0020018.s003.doc]

**Table S2**. Statistical comparison of abundance of Tydeidae, Diptilomiopidae and Phytoseiidae on elms treated with imidacloprid and untreated trees in New York and Maryland.

|  |  | **New York** | | | **Maryland** | | |
| --- | --- | --- | --- | --- | --- | --- | --- |
|  | **Year** | ***F* value** | **df** | ***P* value** | ***F* value** | **df** | ***P* value** |
| **Tydeidae** | 2005 | 0.37 | 1,89 | 0.55 | 0.21 | 1,85 | 0.649 |
|  | 2006 | 40.16 | 1,54 | 0.001^U^ | 6.55 | 1,53 | 0.013^U^ |
|  | 2007 | 32.14 | 1,72 | 0.001^U^ | 21.60 | 1,54 | 0.001^U^ |
| **Diptilomiopidae** | 2005 | 1.12 | 1,89 | 0.304 | 0.37 | 1,85 | 0.544 |
|  | 2006 | 1.47 | 1,54 | 0.241 | 9.59 | 1,53 | 0.001^I^ |
|  | 2007 | 0.99 | 1,72 | 0.334 | 4.69 | 1,54 | 0.001^I^ |
| **Phytoseiidae** | 2005 | 0.84 | 1,89 | 0.361 | 0.28 | 1,85 | 0.596 |
|  | 2006 | 1.54 | 1,54 | 0.214 | 21.67 | 1,53 | 0.001^U^ |
|  | 2007* | 8.86 | 1,72 | 0.004^U^ | 6.84 | 1,54 | 0.012^U^ |

^U^Abundance greater on untreated trees

^I^Abundance greater on imidacloprid-treated trees

Asterisk marks significant time by treatment interactions for Phytoseiidae in NY (*F*_3,72_=3.06, *P*=0.034). Time by treatment interactions were not significant for all other comparisons.
