# Supplementary material for: Neonicotinoid Insecticide Imidacloprid Causes Outbreaks of Spider Mites on Elm Trees in Urban Landscapes
Source: PLoS One. 2011 May 31;6(5):e20018. doi: 10.1371/journal.pone.0020018 (PMC3104998; doi:10.1371/journal.pone.0020018)
Supplement: Table S3 — Comparisons of abundance (number/cm2) of Eriococcidae on elms treated with imidacloprid and untreated elms in Maryland. (DOC) [file pone.0020018.s004.doc]

**Table S3**. Comparisons of abundance (number/cm^2^) of *Gossyparia spuria* on elms treated with imidacloprid and untreated elms in Maryland.

| **Date** | **Test** | **df** | **P value** | **Average (± s.e.m.)**  **Imidacloprid elms** | **Average ± (s.e.m.)**  **Untreated elms** |
| --- | --- | --- | --- | --- | --- |
| 6/15/05 | χ2 = 0.17 | 1 | 0.683 | 0.04 (**±**0.017) | 0.02 (**±**0.008) |
| 6/27/05 | χ2 = 2.98 | 1 | 0.084 | 0.03 (**±**0.011) | 0.02 (**±**0.015) |
| 7/12/05 | F =0.48 | 1,18 | 0.496 | 0.01 (**±**0.003) | 0.02 (**±**0.005) |
| 8/15/05 | χ2 = 3.72 | 1 | 0.054 | 0 (**±**0) | 0.02 (**±**0.012) |
| 9/01/05 | χ2 = 2.35 | 1 | 0.126 | 0 (**±**0) | 0.01 (**±**0.005) |
| 6/14/06 | χ2 = 10.18 | 1 | 0.001 | 0.02 (**±**0.007) | 0.04 (**±**0.136) |
| 7/31/06 | F =74.55 | 1,18 | <0.001 | 0.01 (**±**0.004) | 0.19 (**±**0.031) |
| 9/19/06 | χ2 = 15.25 | 1 | <0.001 | 0 (**±**0) | 0.14 (**±**0.046) |
| 6/05/07 | χ2 = 9.38 | 1 | 0.002 | 0.01 (**±**0.003) | 0.18 (**±**0.069) |
| 6/27/07 | χ2 = 9.17 | 1 | 0.003 | 0.01 (**±**0.005) | 0.16 (**±**0.048) |
| 7/23/07 | χ2 = 7.21 | 1 | 0.007 | 0.02 (**±**0.009) | 0.16 (**±**0.066) |

Non-parametric Kruskal-Wallis tests (χ*^2^*) were used to compare abundance of Eriococcidae when assumptions of ANOVA could not be met through transformations of the data.
