# Supplementary material for: Neonicotinoid Insecticide Imidacloprid Causes Outbreaks of Spider Mites on Elm Trees in Urban Landscapes
Source: PLoS One. 2011 May 31;6(5):e20018. doi: 10.1371/journal.pone.0020018 (PMC3104998; doi:10.1371/journal.pone.0020018)
Supplement: Table S4 — Species scores were generated by PRC analysis to examine responses of individual taxa to imidacloprid applications. (DOC) [file pone.0020018.s005.doc]

**Table S4.** Species scores generated by PRC analysis to examine responses of individual taxa to imidacloprid applications.

|  | **Taxon** | **Score** |
| --- | --- | --- |
| **New York** | Tetranychidae | 3.231 |
|  | Diptilomiopidae | 0.276 |
|  | Phytoseiidae | 0.272 |
|  | Chrysopidae | 0.012 |
|  | Saproglyphidae | 0.003 |
|  | Cecidomyiidae | -0.002 |
|  | Coccinellidae | -0.006 |
|  | Eriococcidae | -0.204 |
|  | Aphididae | -0.232 |
|  | Tydeidae | -0.56 |
| **Maryland** | Tetranychidae | 2.566 |
|  | Diptilomiopidae | 0.635 |
|  | Saproglyphidae | 0.001 |
|  | Coccinellidae | 0.001 |
|  | Chrysopidae | -0.001 |
|  | Thripidae | -0.022 |
|  | Cecidomyiidae | -0.042 |
|  | Aphididae | -0.074 |
|  | Phytoseiidae | -0.397 |
|  | Tydeidae | -0.867 |
|  | Eriococcidae | -1.76 |

Species with scores ≥0.5 and ≤-0.5 contributed significantly to arthropod community response.
