# Supplementary material for: Neonicotinoid Insecticide Imidacloprid Causes Outbreaks of Spider Mites on Elm Trees in Urban Landscapes
Source: PLoS One. 2011 May 31;6(5):e20018. doi: 10.1371/journal.pone.0020018 (PMC3104998; doi:10.1371/journal.pone.0020018)
Supplement: Table S5 — Comparison of feeding rates of S. punctillum and C. rufilabris exposed to spider mites that consumed foliage from imidacloprid-treated elms and untreated elms. (DOC) [file pone.0020018.s006.doc]

**Table S5**. Statistical comparison of feeding rates of *S. punctillum* and *C. rufilabris* exposed to *T. schoenei* that consumed foliage from imidacloprid-treated elms and untreated elms. Time is the duration of exposure to prey.

|  | ***S. punctillum*** | | | ***C. rufilabris*** | | |
| --- | --- | --- | --- | --- | --- | --- |
| **Time** | ***F* value** | **d.f.** | ***P* value** | ***F* value** | **d.f.** | ***P* value** |
| 0.5 h | 2.56 | 1,12 | 0.136 | 35.34 | 1,12 | 0.001 |
| 1.5 h | 20.34 | 1,12 | 0.001 | 42.03 | 1,12 | 0.001 |
| 2.5 h | 58.36 | 1,12 | 0.001 | 44.06 | 1,12 | 0.001 |
