# Supplementary material for: Neonicotinoid Insecticide Imidacloprid Causes Outbreaks of Spider Mites on Elm Trees in Urban Landscapes
Source: PLoS One. 2011 May 31;6(5):e20018. doi: 10.1371/journal.pone.0020018 (PMC3104998; doi:10.1371/journal.pone.0020018)
Supplement: Table S6 — Comparison of mobility of S. punctillum and C. rufilabris exposed to imidacloprid in prey and foliage. (DOC) [file pone.0020018.s007.doc]

**Table S6**. Comparison of mobility (√mm/s) of *S. punctillum* and *C. rufilabris* exposed to imidacloprid in prey and foliage.

|  |  | ***S. punctillum*** | | | | ***C. rufilabris*** | | | |
| --- | --- | --- | --- | --- | --- | --- | --- | --- | --- |
| **Time** | **Treatment** | **Average (±s.e.m.)** | ***F* value** | **df** | ***P* value** | **Average (±s.e.m.)** | ***F* value** | **df** | ***P* value** |
| 0.5 h | Untreated mites | 1.65 (± 0.09) **a** | 36.93 | 3,24 | 0.001 | 1.77 (± 0.13) **ab** | 9.52 | 3,24 | 0.001 |
|  | Imidacloprid mites | 0.76 (± 0.04) **b** |  |  |  | 0.95 (± 0.1) **d** |  |  |  |
|  | Untreated leaves | 1.84 (± 0.09) **a** |  |  |  | 1.55 (± 0.14) **abc** |  |  |  |
|  | Imidacloprid leaves | 1.79 (± 0.1) **a** |  |  |  | 1.65 (± 0.11) **ab** |  |  |  |
| 1.5 h | Untreated mites | 1.72 (± 0.11) **a** | 20.99 | 3,24 | 0.001 | 1.8 (± 0.11) **ab** | 15.26 | 3,24 | 0.001 |
|  | Imidacloprid mites | 0.75 (± 0.04) **b** |  |  |  | 0.92 (± 0.07) **d** |  |  |  |
|  | Untreated leaves | 1.75 (± 0.14) **a** |  |  |  | 1.77 (± 0.15) **ab** |  |  |  |
|  | Imidacloprid leaves | 1.69 (± 0.11) **a** |  |  |  | 1.86 (± 0.13) **a** |  |  |  |
| 2.5 h | Untreated mites | 1.51 (± 0.09) **a** | 19.65 | 3,24 | 0.001 | 1.73 (± 0.11) **ab** | 8.1 | 3,24 | 0.001 |
|  | Imidacloprid mites | 0.8 (± 0.07) **b** |  |  |  | 1.0 (± 0.13) **d** |  |  |  |
|  | Untreated leaves | 1.79 (± 0.12) **a** |  |  |  | 1.7 (± 0.1) **ab** |  |  |  |
|  | Imidacloprid leaves | 1.62 (± 0.11) **a** |  |  |  | 1.67 (± 0.14) **ab** |  |  |  |
| 3.5 h | Untreated mites | 1.65 (± 0.11) **a** | 11.86 | 3,24 | 0.001 | 1.54 (± 0.1) **ab** | 14.55 | 3,24 | 0.001 |
|  | Imidacloprid mites | 0.9 (± 0.08) **b** |  |  |  | 0.8 (± 0.09) **d** |  |  |  |
|  | Untreated leaves | 1.62 (± 0.13) **a** |  |  |  | 1.61 (± 0.11) **ab** |  |  |  |
|  | Imidacloprid leaves | 1.66 (± 0.15) **a** |  |  |  | 1.31 (± 0.06) **bcd** |  |  |  |

Predators were exposed to 1) *T. schoenei* that consumed foliage from imidacloporid-treated elms and untreated elms (Untreated mites; Imidacloprid mites) and 2) leaves without *T. schoenei* (Untreated leaves; Imidacloprid leaves). Means within each time point with different letters are statistically different at *P*<0.05 (Tukey’s test).
