# Supplementary material for: Neonicotinoid Insecticide Imidacloprid Causes Outbreaks of Spider Mites on Elm Trees in Urban Landscapes
Source: PLoS One. 2011 May 31;6(5):e20018. doi: 10.1371/journal.pone.0020018 (PMC3104998; doi:10.1371/journal.pone.0020018)
Supplement: Table S7 — Comparison of nitrogen levels in elm trees treated with imidacloprid in New York (NY) and Maryland (MD) in 2005 (DOC) [file pone.0020018.s008.doc]

**Table S7**. Comparison of nitrogen levels in elm trees treated with imidacloprid in New York and Maryland in 2005.

|  |  | **New York** | | | | **Maryland** | | | |
| --- | --- | --- | --- | --- | --- | --- | --- | --- | --- |
|  | **Treatment** | **% Nitrogen (± s.e.m)** | ***F* value** | **df** | ***P* value** | **% Nitrogen (± s.e.m)** | ***F* value** | **df** | ***P* value** |
| **June** | Untreated | 2.65 (± 0.1) | 0.05 | 1,18 | 0.832 | 1.94 (± 0.03) | 0.11 | 1,18 | 0.744 |
|  | Imidacloprid | 2.62 (± 0.1) |  |  |  | 1.96 (± 0.04) |  |  |  |
| **August** | Untreated | 1.97 (± 0.06) | 4.17 | 1,18 | 0.056 | 1.95 (± 0.02) | 0.23 | 1,18 | 0.641 |
|  | Imidacloprid | 2.19 (± 0.09) |  |  |  | 1.97 (± 0.03) |  |  |  |
