# Supplementary material for: Neonicotinoid Insecticide Imidacloprid Causes Outbreaks of Spider Mites on Elm Trees in Urban Landscapes
Source: PLoS One. 2011 May 31;6(5):e20018. doi: 10.1371/journal.pone.0020018 (PMC3104998; doi:10.1371/journal.pone.0020018)
Supplement: Table S8 — The number of trees in different size classes treated by different methods of application for 2005, 2006, and 2007. (DOC) [file pone.0020018.s009.doc]

**Table S8.** The number of trees in different size classes treated by different methods of application for 2005, 2006, and 2007.

|  |  | **Tree diameter (cm^2^)** | | | | | | | | | | | | | | | | |
| --- | --- | --- | --- | --- | --- | --- | --- | --- | --- | --- | --- | --- | --- | --- | --- | --- | --- | --- |
| **Year** | **Application** | **0-10** | **11-20** | **21-30** | **31-40** | **41-50** | **51-60** | **61-70** | **71-80** | **81-90** | **91-100** | **101-110** | **111-120** | **121-130** | **131-140** | **141-150** | **181-190** | **291-300** |
| 2005 | trunk injection | 2 | 144 | 1089 | 37 | 32 | 1 | 10 | 6 | 2 | 0 | 0 | 0 | 0 | 0 | 0 | 0 | 0 |
| 2005 | soil drench | 0 | 0 | 0 | 2 | 0 | 0 | 26 | 0 | 0 | 0 | 0 | 0 | 0 | 0 | 0 | 0 | 0 |
| 2005 | soil injection | 11 | 6 | 162 | 385 | 863 | 1071 | 707 | 217 | 11 | 5 | 0 | 0 | 0 | 0 | 0 | 0 | 0 |
| 2006 | trunk injection | 9 | 182 | 1159 | 83 | 6 | 0 | 0 | 6 | 3 | 0 | 0 | 0 | 0 | 0 | 0 | 0 | 0 |
| 2006 | soil drench | 0 | 0 | 0 | 0 | 6 | 0 | 5 | 0 | 0 | 0 | 0 | 1 | 0 | 0 | 0 | 0 | 0 |
| 2006 | soil injection | 1 | 1 | 34 | 210 | 1199 | 914 | 786 | 255 | 1 | 0 | 0 | 0 | 0 | 0 | 0 | 0 | 0 |
| 2007 | trunk injection | 28 | 84 | 48 | 46 | 26 | 12 | 29 | 6 | 13 | 2 | 3 | 0 | 0 | 0 | 1 | 3 | 0 |
| 2007 | soil drench | 289 | 727 | 383 | 526 | 631 | 505 | 1032 | 347 | 189 | 14 | 2 | 44 | 1 | 2 | 1 | 0 | 23 |
